# Phyre2.2: A community resource for template-based protein structure prediction

**DOI:** 10.1016/j.jmb.2025.168960

**Published:** 2025-01-23

**Authors:** Harold R Powell, Suhail A Islam, Alessia David, Michael J E Sternberg

**Affiliations:** 1 Department of Life Sciences, Imperial College London, London SW72AZ, UK; 2 Department of Life Sciences, Imperial College London, London SW72AZ, UK; 3 Department of Life Sciences, Imperial College London, London SW72AZ, UK; 4 Department of Life Sciences, Imperial College London, London SW72AZ, UK

## Abstract

Template-based modelling, also known as homology modelling, is a powerful approach to predict the structure of a protein from its amino acid sequence. The approach requires one to identify a sequence similarity between the query sequence and that of a known structure as they will adopt a similar conformation, and the known structure can be used as the template for modelling the query sequence. Recently several approaches, most notably AlphaFold, have employed enhanced machine learning and have yielded accurate models irrespective of whether there is an identifiable template. Here we report Phyre2.2 which incorporates several enhancements to our widely-used template modelling portal Phyre2. The main development is facilitating a user to submit their sequence and then Phyre2.2 identifies the most suitable AlphaFold model to be used as a template. In Phyre the user searches a template library of known structures. We have now included in our library a representative structure for every protein sequence in the protein databank (PDB). In addition, there are representatives for an apo and a holo structure if they are in the PDB. The ranking of hits has been modified to highlight to the user if there are different domains spanning the sequence. Phyre2.2 continues to support batch processing where a user can submit up to 100 sequences facilitating processing of proteomes. Phyre2.2 is freely available to all users, including commercial users, at https://www.sbg.bio.ic.ac.uk/phyre2/.

## Graphical abstract

**Figure:**
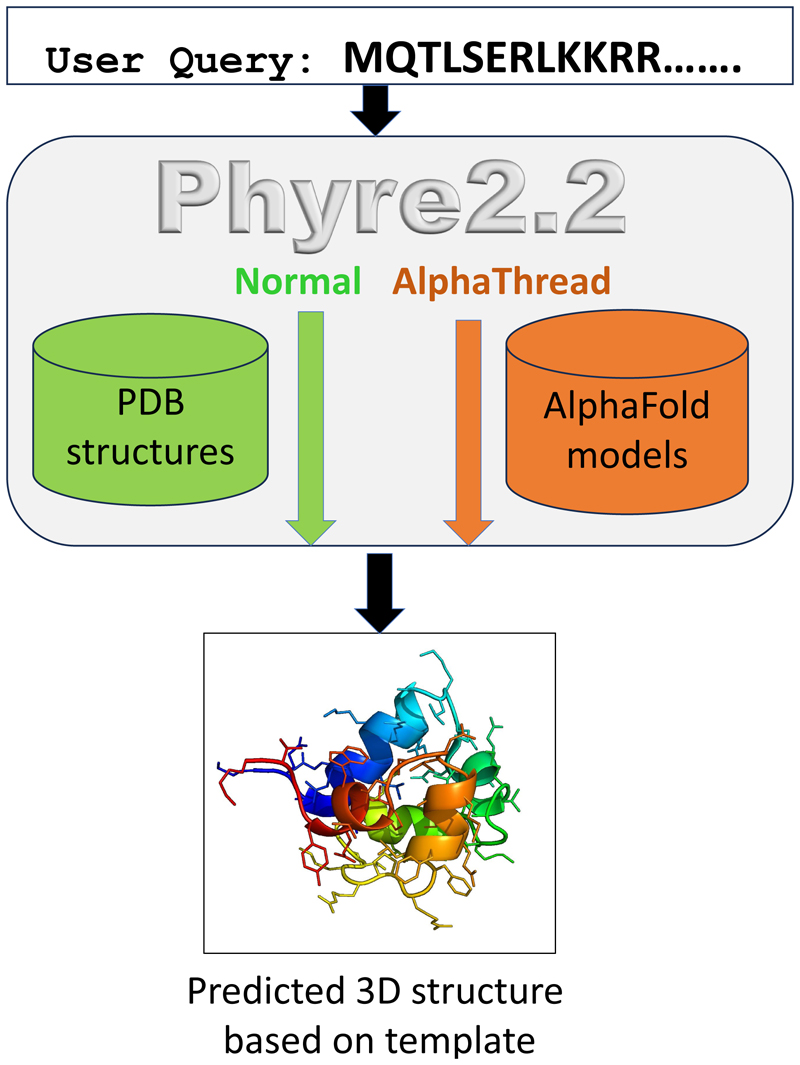

## 1 Introduction

Sequences, often including those of entire proteomes, are being determined at an ever-increasing rate. Consequently, many researchers in the bioscience and biomedical communities are looking for models of proteins to provide insight into structure/function relationships and to guide further experiments. For many years template-based modelling, also known as homology modelling, has been the most accurate approach for structure prediction and has been made accessible to the community via web servers including Phyre2^1^ and SWISS-Model^2^. The result of a sequence search identifies a sequence similarity between the query to be predicted and a known structure from which one infers that the unknown structure will adopt a similar conformation to the known coordinates which accordingly acts as a template for modelling. In the absence of a viable template, template-free approaches have employed several strategies including assembling fragments into a fold (e.g. I-TASSER^3^ and ROSETTA^4^) and the use of co-evolution of residues to infer residue proximity (e.g. ref ^5^ ). These template-free methods can yield good models for many proteins, but often these are not of the same quality as the models obtained by template-based modelling.

Recently the development of prediction using advanced machine learning, most notably the AlphaFold3^6^ and AlphaFold2^7^ approaches but also others including RoseTTAfold^8^ and ESMFold^9^, have revolutionised structure prediction, often yielding highly accurate models for queries irrespective of whether there is a known template. Models predicted by AlphaFold2 for over 200 million sequences in the UniProt database are available from the European Bioinformatics Institute (EBI)^10^. Moreover, users can model their own sequence using AlphaFold2 and AlphaFold3 by installing code in-house or can run AlphaFold2 via the ColabFold server^11^. In addition, the community can access predicted structures for more than 1 million sequences at the Research Collaboratory for Structural Biology (RCSB)^12^ as they provide models generated by several approaches, particularly by AlphaFold2 and RoseTTAfold. Users can run RoseTTAfold via a web server and downloadable code. There are also models for over 700 million metagenomic sequences available from MetaAI’s web site https://esmatlas.com/. However, in general, models from AlphaFold3, AlphaFold2 and RoseTTAfold, which employ multiple sequence alignment, are more accurate than those from ESMFold, which are based on language models. All these developments have markedly expanded the number of structures that can be used for template-based modelling.

For over 20 years, our group has been providing the community with template-based modelling via our Phyre/Phyre2 servers^1^. These servers were designed to meet the needs of the broad community, many of whom are not experts in structural bioinformatics. Due to its ease of use, facilities for batch processing together with other features, Phyre2 has been extensively used, with over 10,000 citations to the 2015 paper describing the resource. Despite the availability of many deposited predicted models and the opportunity to run AlphaFold on any sequence, Phyre2 continues to be used with over 38,250 unique users and over 900 citations in calendar year 2024. This demonstrates that an easy-to-use template modelling server continues to have major value to the community. Firstly, it provides an alternative approach that can assist in assessing a model generated by these recent deep learning approaches such as AlphaFold. In particular, it can provide a model based on a user-defined single known template, for example a user-defined apo or holo structure. Simply using the deposited AlphaFold structure often does not provide a user with the opportunity to have a model that represents either the apo or the holo structure. Secondly, it provides an easy approach to model a sequence whose structure is not available but there is an appropriate experimental or predicted template.

This paper reports Phyre2.2 which is the next generation of our widely-used template modelling web server Phyre2. A key enhancement is facilitating the user to input a sequence, perform a Blastp^13^ search to find the closest AlphaFold2 structure at the EBI database, and generate a model. A similar procedure is available at SWISS-Model^2^. Other developments include (i) a new template library that covers each UniProt sequence in the Protein Data Bank (PDB)^12,14^ deposited coordinates including, if available, one representative for the apo and holo coordinates, and (ii) an enhanced approach for a user to easily identify models for different domains within the query sequence. Phyre2.2 is available to all, including commercial users, at https://www.sbg.bio.ic.ac.uk/phyre2/.

## 2 Phyre2.2 in normal mode

### Template library construction

**Figure 1:**
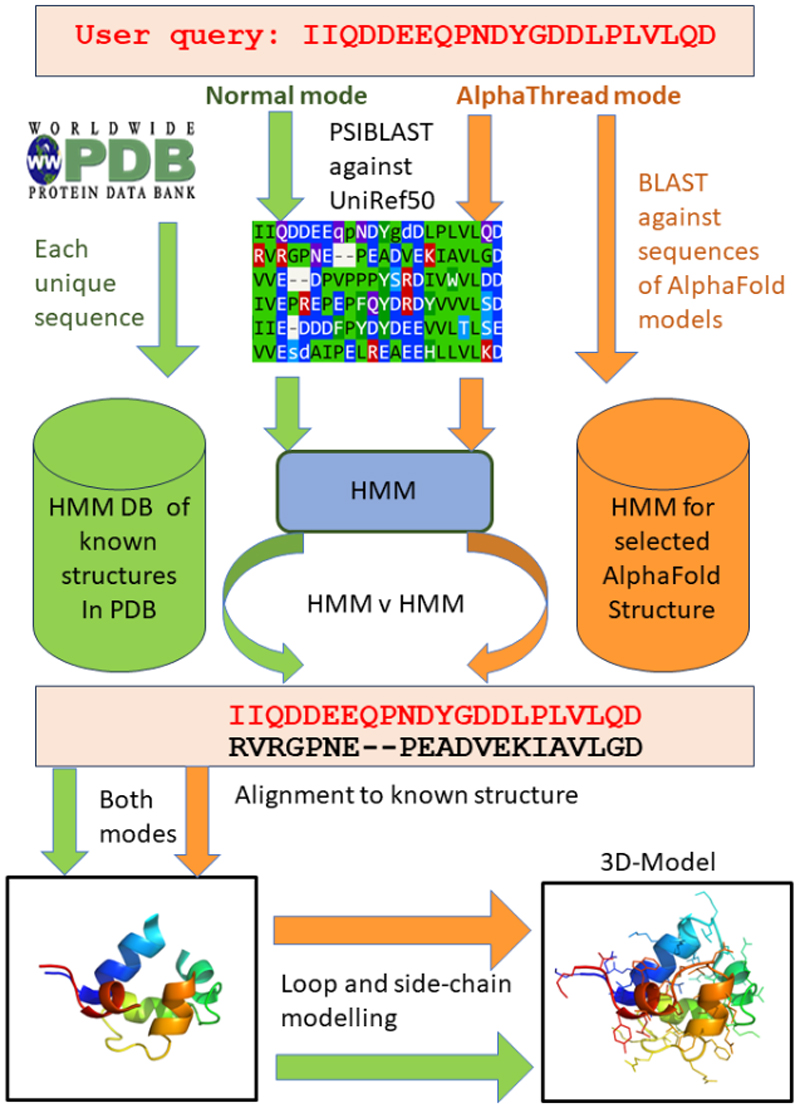
Flow chart of Phyre2.2 in normal (left) and AlphaThread (right) modes. Starting on the top left, the approach of generating an HMM from the query sequence and matching it again a library of HMMs for known structures using HHBlits is shown. Starting on the top right, the approach of searching the query sequence against the sequences in the EBI library of AlphaFold predicted structures is illustrated. Once templates (for normal mode) or the AlphaFold template (for AlphaThread) is identified the figure shows the construction of the model involving loop modelling and side chain placement.

Figure 1 describes the standard modelling approach used by Phyre2.2 which we term “normal mode”. Here we report the entire process highlighting changes since our earlier paper. A new feature in Phyre2.2 is its template library. In Phyre2, the template library was selected to be representative, so no entry had more than 70% sequence identity to another. This facilitated faster searching on the hardware available at the time. In Phyre2.2, we now include coordinates for each UniProt sequence with a PDB entry and, if available, sets of coordinates for both apo and holo templates. If there are different PDB files covering different segments of the UniProt sequence^14^, then each covered segment is included in the library. In addition, the PDB contains several entries that are not associated with a UniProt entry. These include antibodies, viral proteins, and engineered proteins. We include in the template library representatives of these non-UniProt entries where they share no more than 75% protein sequence identity. In selecting which PDB entry to take when there were alternatives to cover the sequence we used the metric of model quality “rankingScore” from the UniProt json files calculated by the Protein Data Bank in Europe (PDBe) and available at https://www.ebi.ac.uk/pdbe/graph-api/uniprot/unipdb/ for UniProt accessions associated with PDB entries. As of 1^st^ October 2024 there were approximately 6000 PDB entries that did not have an associated UniProt accession. Our revised template library will enable Phyre2.2 to use a close homologue to a query sequence which should facilitate generating a high-quality model. Moreover, a user will be able to select a model based on either an apo or a holo template, if available.

The sequence of each entry in the template library is selected in turn and using PSI-Blast ^13^ scanned against UniRef50^15^ to generate a multiple sequence alignment. The known secondary structure is assigned from the coordinates using DSSP^16^ and is also predicted using PSIPRED^17^. The multiple sequence alignment together with the known and predicted secondary structures are represented within a hidden Markov model (HMM) generated by HHBlits^18^.

### Query sequence input

Figure S1 shows the landing and input page for Phyre2.2. A user of normal mode provides the title of their search and pastes into the sequence box their one letter sequence in FASTA format (without the title line starting with >). A file with the input sequence can also be uploaded. Sequences are checked to be protein-like and, for example, we trap if a user submits a nucleotide sequence. Alternatively, a user can supply a UniProt code and the sequence and title boxes are populated. In both modes, the user supplies their e-mail where we will send a link to their results. The expected turnaround time on submission is indicated and typically is a few hours.

### Matching query sequence to template

A similar procedure to that used to generate the HMM for the template is applied to the query sequence. The query sequence is scanned against the UniRef50 library of sequences using PSI-Blast and a multiple sequence alignment generated. The secondary structure is predicted using PSIPRED. The multiple sequence alignment together with the predicted secondary structure is combined using HHBlits to generate the HMM. The HMM for the query is scanned in turn against each HMM in the template library using HHBlits and a list of matches generated. Each match returns a sequence alignment of the query with the template together with the associated E-value (Expectation value) and the percent sequence identity within the alignment. Except for orphan sequences, HMM /HMM matching is expected to yield a more accurate alignment than obtained via Blastp. Moreover HMM/HMM matching can identify more remote homologues than Blastp. In the earlier version of Phyre2 the slower HHSearch algorithm^18^ was used for HMM/HMM searching.

### Loop modelling

Where there is an alignment match of a query and a template residue, the backbone coordinates of the template are retained. From the alignment, indels (insertions / deletions) in the alignment are identified and here loop modelling is required. As in Phyre2, a library of fragments in length ranging from 2 to 15 residues is extracted from the PDB. A search is now conducted for fragments that (i) have a similar sequence to the residues in the indel and (ii) can be melded onto the optimal set of a few residues that define the framework before and after the break. The selected fragment is then melded onto the template framework.

### Side-chain modelling

The final step in normal mode is to replace the side chains in the template and the fitted fragments with those of the query sequence. We replaced the earlier approach for side-chain replacement with SCWRL4^19^. This searches through a backbone-dependent library of side-chain rotamers for a packing that has optimal hydrogen bonding and van der Waals packing.

### Ranking of hits returned by HHBlits

Prior to 2023, only the E-value returned by HHBlits was used to rank the hits with the lowest E-value at the top of the list. As a consequence of how E-values are estimated in HHBlits, this sometimes led to a model with the lowest E-value being placed higher in the list compared to a model which had a slightly larger E-value but with a far higher sequence identity to the template but covering only slightly fewer residues of the query. In 2023, we introduced a new heuristic algorithm that ranked the hits to consider both the E-value return by HHBlits and the percent sequence identity between the matched section of the query and the template. Our heuristic algorithm balances sequence identity and E-value returned by HHBlits promoting the higher ranking of matches with higher sequence identity (see Supplementary Data). Another improvement to the ranking of hits is to place models that cover different sections of the query high in the list irrespective of their rank order (see Supplementary Data). This ensures that if different domains in the protein could be modelled, the predictions for the different domains are placed towards the top of the list and thus the presence of these models is rapidly identifiable to the user.

### Output of predicted structures

**Figure 2:**
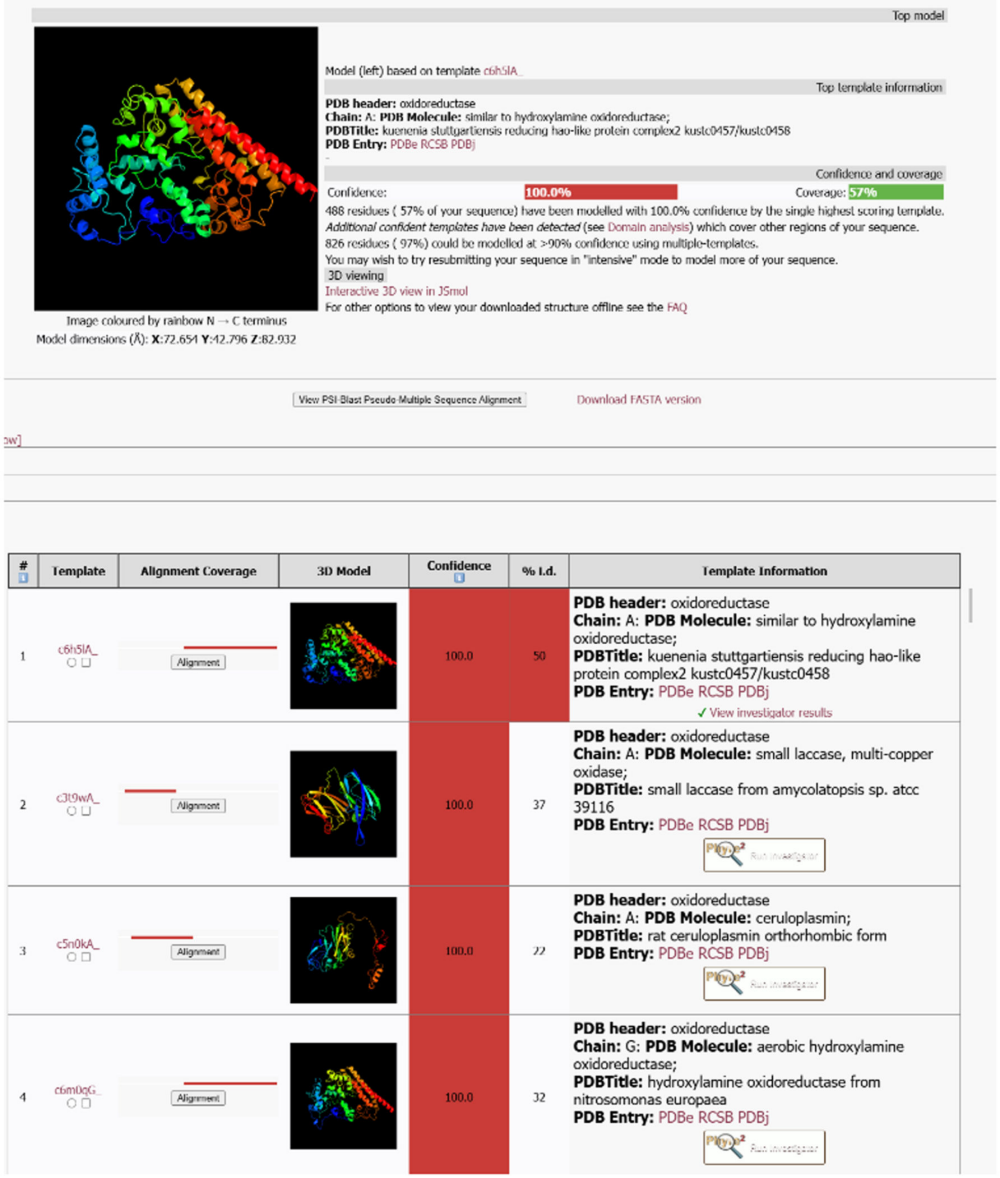
The output from a run of Phyre2.2 in normal mode The query is UniProt entry P0DV45 and the output shows how the first hit provides a model for the C-terminal region and the second hit for the N-terminal domain. Clicking on the images enables the user to download the coordinates of the predicted model.

The top of main output page when Phyre2.2 is run in normal mode presents an image of the top ranked hit which can be viewed using JSMol^20^ (Figure 2). The page reports the PDB code of the template used together with confidence that this template used is a true homologue and therefore the fold is correctly predicted. This confidence is obtained from the E-value of HHBlits. It is important to note that this is not the confidence that every residue is correctly placed. In addition, the percentage coverage by the model of the query sequence is reported.

Scrolling down the output, Phyre2.2 presents a list of the top 20 matches with their confidence values colour coded according to confidence range together with the percent sequence identity between the aligned query residues and the template sequence. These 20 hits, which include the top hit, have all been modelled and images presented. As with the top model, an image can be viewed via JSMol^20^ and the coordinates downloaded. There is also a linear image showing the section of the query sequence that was modelled. When the user clicks on the box “alignment”, a new window opens and displays the alignment of the query and template together with the predicted secondary structure of the query and the predicted and actual secondary structures of the template (Figure S2). Inspection of the alignment for equivalenced functional residues is often a powerful approach to select the best model from a set of similarly scoring matches. To aid this, functional residues in the template obtained from M-CSA^21^ (Mechanism and Catalytic Site Atlas) are highlighted. After these top 20 hits, a further 100 hits are presented as above with the exception that these templates were not used for modelling and no structure is presented. If a user wishes to build the model upon this template, they can use one-to-one threading as described below.

### Additional information provided

The main output page has links to additional information. The PSI-Blast alignment of the query can be viewed and downloaded in FASTA format. The predicted secondary structure using PSIPRED and predicted disordered regions using DisoPred^22^ of the template are presented together with confidence indications; a pdf image suitable for publication can be downloaded. The link to domain analysis provides a linear representation of the templates that match sections of the query sequence. Mouse-over any of the top 20 hits provides a summary of the results including an image of the predicted structure. Scrolling down this domain analysis is particularly helpful to identify if a further section of the query sequence is covered in addition to the top few hits. A pdf image of this display can be downloaded.

### Phyre Investigator

For each of the top 20 models, there is a link to initiate further analysis of the structure by Phyre Investigator. This takes about five to ten minutes to run. The model is displayed and selected features colour coded to indicate the results of the analysis. The predicted model is analysed for model quality using various metrics including ProQ2^23^, clashes, disallowed Ramachandran (ϕ,φ) backbone torsions, alignment quality and disordered regions. Insights into function are provided by analysing sequence conservation and the detection by fpocket2^24^ of pockets suitable to include a ligand.

## 3 AlphaThread via one-to-one threading

Figure 1 also illustrates the new feature AlphaThread in Phyre2.2 which enables a user to identify an AlphaFold2 model^10^ whose sequence is closest to the query and to use this as a template. On the input page the user selects AlphaThread (Figure S1). Blastp^13^ is used to find homologous sequences in the AlphaFold database; the coordinates of the top hit are downloaded and one-to-one threading is run for this model. Alternatively, a user can supply the UniProt code of the AlphaFold2 model that will serve as the template. The AlphaFold2 database at the EBI only provides full length predictions for proteins of no more than 2,700 residues. Otherwise, the approach for the AlphaFold database is to segment the sequence into sections of 1,400 and each section is predicted by AlphaFold and deposited in the EBI database. Accordingly, we have limited AlphaThread to only model sequences of no more than 2,700 residues. If a user needs to model longer proteins, they will need to identify the segments of interest in the EBI database and run Phyre2.2 using the one-to-one threading mode as described below. For all models AlphaFold2 provides an evaluation of the accuracy of location of a residue in the structure via the pLDDT (predicted Local Distance Difference Test) metric which ranges from 0 to 100, with 100 representing the highest level of confidence. In keeping with standard practice, residues with a pLDDT of less than 70 are considered unreliable and are removed from the model before further analysis^7^. Having identified the AlphaFold2 template, Phyre2.2 follows one-to-one threading. An HMM is constructed for the query and for the selected template as in normal mode and HHSearch (rather than HHBlits) is currently used to align the sequences. Once the alignment is obtained, the procedure for loop fitting and side-chain modeling follows that in Phyre2.2 normal mode. If a segment of the AlphaFold model has a run of pLDDT< 70 for no more than 15 residues, this will have been removed and then Phyre2.2 will undertake its standard loop modelling procedure. Longer loops excised from the AlphaFold structure are not modelled in Phyre2.2. As there is only one template, the output consists of the alignment and the model (Figure S3).

More generally, in one-to-one threading, the user uploads their sequence and the coordinates of their selected single-chain template in PDB format and then the above protocol is followed: generate the HMMs, align using HHSearch, loop modeling and side-chain placement. (We plan to replace HHSearch with HHBlits, but as only a single alignment if performed this has trivial impact on performance.) The output format is the same as returned when a user runs AlphaThread.

## 4 Additional features in Phyre2.2

Phyre2.2 retains the several of original features of Phyre2 and we report these in outline for completeness.

### Intensive mode

This mode (Figure S4) can be selected by a radio button on the front input page and is used to generate a complete model when different sections of the query sequence are covered by different templates. A heuristic defines the components to meld into the final model. Our procedure Poing^25^ uses linear springs as constraints between Cα atoms. The protein is modelled by synthesis from its N terminus as if folding off a ribosome and structures are generated that avoid steric clashes. These predicted structures are clustered, and the most populated cluster selected as the final model. The intensive mode can be highly effective if there is an overlap of residues between the different segment models, but if there is a large gap generally the link between the sections is poorly modelled. However, it can be useful to view the rough relative positions of the different sections that have been modelled under normal mode. The confidence of residues modelled are represented by a colour coding. Intensive mode should only be used once a user has run normal mode and several domains identified in the output.

### Batch processing

This facility enables a user to upload a file with up to 100 sequences in FASTA format and run the normal mode modelling in the background when computing power is available. The results are summarised in a table with links to each model and the results can be downloaded. The maximum number of sequences can be increased on request to the program’s authors. With more entire proteomes being sequenced, this facility is widely used and accounts for nearly 60% of all Phyre2.2 jobs.

### PhyreAlarm

The model generated by Phyre depends on the availability of a template. The PDB is updated weekly and so a template may be available for a sequence (or sequence segment) that was previously unmodelled. Additionally, even if the query is modelled, a structure with higher sequence identity (quantified by the confidence value returned by HHBlits) may have been deposited after a previous run and this is expected to yield a more accurate model. In PhyreAlarm, the user can simply request weekly scans against any newly deposited structure in the template library. If there is a match against the newly deposited template structures that is expected to yield a better model, the query sequence is run through normal mode to generate a model and the user notified by e-mail. If no new match is found, the request is stored and rerun the next week without additional user intervention.

### BackPhyre

As its name suggests, this starts with a structure uploaded by the user and searches a library of proteomes to detect if a similar structure exists. Currently there are the sequences of 51 proteomes and a user can request further proteomes to be added if required. The uploaded structure is processed to generate an HMM using the same approach as used to generate the HMM for the template library. Each sequence in the proteomes is processed as the query sequence in Phyre normal mode to yield an HMM. Then HMM/HMM searching is performed and matches reported.

### GenSearch

This feature enables a user to use HMM/HMM searching against a library of proteomes. HMMs have been generated for sequences in our library of proteomes as for a query in normal mode. Similarly, an HMM is generated for the user supplied query sequence. HMM matching identifies sequences matches. Click on a hit (on the i button) provides an alignment of the sequences together with their predicted secondary structures.

### Job manager

The results of a search are kept for 30 days. Before they are deleted, you can request the results to be kept for a further 30 days. The job manager, which is available for users who have registered, enables you to view all jobs submitted under your e-mail and to view the results if still on the system.

### User support

In addition to documentation, Phyre2.2 supports a Google group for discussions from the community and a X-feed to notifications.

## 5 Examples

As an example, we show the results of submitting the 852 residue UniProt reviewed entry P0DV45 which is a “Probable nitrite reductase-hydroxylamine oxidoreductase fusion protein” from bacterium *Kuenenia stuttgartiensis*. Figure 2 shows the results of the normal search with the top hit covering residues 350 – 848 with the aligned region having 50% protein sequence identity to the template. With the new sorting algorithm, the second hit covers a different domain spanning 36-311 with a sequence identity of 37% to the template. Both matches show a confidence of 100% which is the probability that a homologous template is being used. The alignment is shown in Figure S2. The output when intensive mode is run is shown in Figure S4.

There is no AlphaFold predicted structure in the EBI database for P0DV45 and Figure S3 shows the top and bottom of the output running AlphaThread. At the top the selected template from the AlphaFold database at the EBI is indicated and the bottom has the predicted structure displayed.

**Figure 3:**
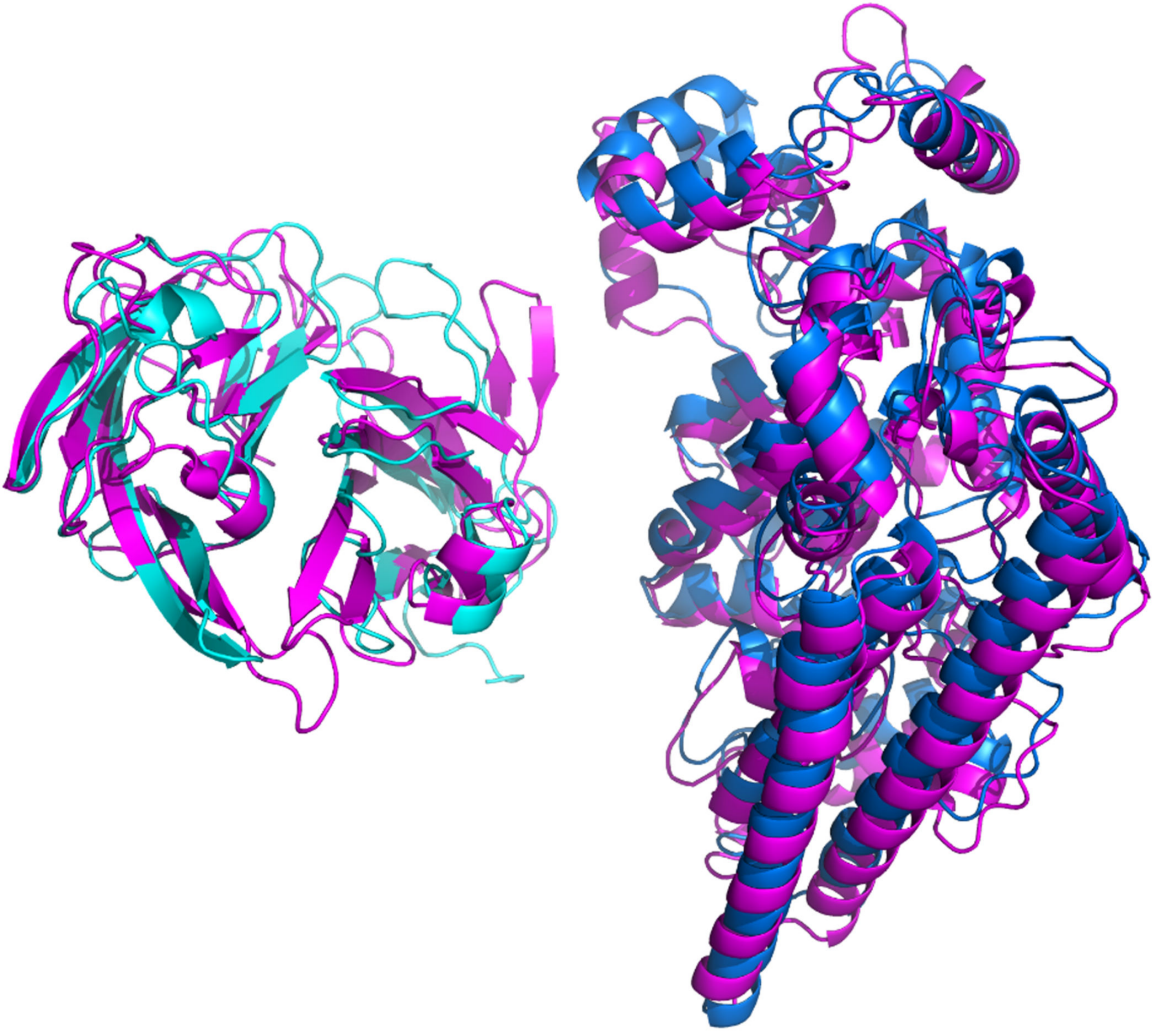
Superposition of the predicted structure of UniProt P0DV45 from normal mode and AlphaThread. UniProt P0DV45 is a “Probable nitrite reductase-hydroxylamine oxidoreductase fusion protein” from bacterium *Kuenenia stuttgartiensis*. The Figure shows the overlap of the top two domains predicted in normal mode with the model generated using AlphaThread. The model from AlphaThread is in magenta. The N-terminal domain (residues 36 – 311) predicted by normal mode is in cyan and the C-terminal domain (residues 350 – 848) is in dark blue. α–helices are denoted by spirals and β-sheet strands as arrows. Figure generated using PyMol^26^.

Figure 3 shows a PyMol^26^ generated image of the overlap of (i) the top two domains predicted in normal mode based on PDB experimental templates with (ii) the model generated using AlphaThread based on an AlphaFold model. The model from AlphaThread is in magenta. The N-terminal domain predicted by normal mode is in cyan and the Cα RMS deviation of the 240 aligned residues is 2.7Å. The Cα RMS for the 492 aligned residues in the C-terminal is 2.2 Å. Thus, both approaches will yield a similar model although the details will vary.

## 6 Discussion and conclusion

The availability of over 200 million structures predicted by AlphaFold2 and over 700 million by ESM has resulted in a vast set of models that can serve for templates for homology modelling servers such as Phyre and SWISS-Model. Moreover, the number of experimental structures in the PDB is increasing which again results in more available templates. We consider that template-based modelling will now have a particularly valuable role when there is a high degree of sequence similarity between the query and template. With this high sequence similarity, the structure can be expected to be well modelled. Our previous study has benchmarked the accuracy of Phyre modelling and for a sequence identity in the 90-95% identity range, the median root mean square deviation of Cα alpha atoms (Cα rmsd) is 0.86Å, while in the identity range 60%-69% the median Cα rmsd is 1.35Å.

In recognition that a major role of Phyre2.2 will be in modelling close homologues, the template library now has full coverage of every UniProt sequence with an available experimental structure, rather than the representative set employed in Phyre2. Consequently, we expect that Phyre2.2 will generally yield better models than Phyre2 when there is a template with sequence identity above 80% to the query. However, the CAMEO^27^ continuous assessment focusses on the more difficult modelling targets and rarely, if ever, has queries where there is a template with more than 80% identity to the query.

Furthermore, when there is an apo and a holo structure in the PDB, a user will be able to scan the ranked list of hits and from the details of the PDB template select the model that best suits the biological problem they aim to tackle. Additionally, when there is a newly determined sequence that is closely related to a homologue whose structure was predicted by the latest machine learning approaches such as AlphaFold, a user can use one-to-one threading to upload their chosen model. In particular, Phyre2.2 enables a user to identify the closest AlphaFold model and predict a structure for the query sequence. We envisage that one major application of Phyre2.2 is when there is a query sequence (Q) whose structure is not in the AlphaFold database but that of a close homologue (H) is. In general, the 3D model of Q predicted *ab initio* by AlphaFold2 or AlphaFold3 is expected to be more accurate that the homology modelled structure of Q obtained with Phyre2.2 using H as the template. However, if Q and H are close homologues, the difference in the predicted structures is expected to be very small and the Phyre2.2 model should be appropriate for many applications. The ease of use of Phyre2.2 compared to running AlphaFold2 or AlphaFold3, and the additional information provided by Phyre2.2, should be of value to the community. More generally, we envisage that predicting structures via Phyre2.2 can be used to complement predictions by AlphaFold and help to identify the most confidently modelled regions.

We have also developed Phyre2.2 to take into account recent developments in protein bioinformatics and computing. The use of HHBlits has speeded up sequence searching of the template library and a run in normal mode for proteins of lengths 500 and 100 residues typically takes less than half the time taken when using HHSearch^18^. We have extended the limit of the length of the query from 3,000 to 6,000 residues. The load on Phyre2 varies but, given the international use of the resource, during weekdays a user can expect a turnaround in a few hours. A user can submit many jobs at one time, and we do not require one job to finish before another can start.

Although Phyre2.2 is designed to generate a structure based on a closely-relate template, we must emphasise that predicting a wild-type and a mutant structure with a single residue change is most likely to yield virtually the same structure. This cannot be interpreted that the variant is benign in terms of its structural impact. Specific software modelling variants (reviewed in ref^28^ ), such as our Missense3D suite ^29–31^ or FoldX ^32^ should be used for this task.

To summarise, Phyre2.2 is a web portal for template-based protein structure prediction. A major development beyond Phyre2 is that it enables a user to input their sequence and obtain a model based on an AlphaFold2 predicted structure. More generally, the one-to-one threading facility enables a user to base their prediction on any uploaded set of coordinates, including ones from the vast pool of predicted structures. Other developments include a complete coverage of all UniProt sequences in the PDB, with apo and holo coordinates if available. These developments should serve to maintain Phyre2.2 as a valued resource for many users who often will not have expertise in structural bioinformatics.

## Supplementary Material

Supplementary data

## Highlights

- Template-based structure prediction yields accurate models when there is a good sequence similarity between query and template.
- Phyre2.2 is the next generation of our template-based protein structure modelling portal Phyre2.
- Novel to Phyre2.2 is that it enables a user to identify a template from the AlphaFold database and thereby model a newly sequenced protein not previously modelled by AlphaFold.
- Additional enhancements to Phyre2.2 include a template library that facilitates modelling of apo and holo structures and a ranking of hits that highlights models for different domains in the query.

## References

[1] Kelley LA, Mezulis S, Yates CM, Wass MN, Sternberg MJ (2015). The Phyre2 web portal for protein modeling, prediction and analysis. Nature protocols.

[2] Waterhouse A, Bertoni M, Bienert S, Studer G, Tauriello G, Gumienny R, Heer FT, de Beer TAP, Rempfer C, Bordoli L (2018). SWISS-MODEL: homology modelling of protein structures and complexes. Nucleic acids research.

[3] Zhou X, Zheng W, Li Y, Pearce R, Zhang C, Bell EW, Zhang G, Zhang Y (2022). I-TASSER-MTD: a deep-learning-based platform for multi-domain protein structure and function prediction. Nature Protocols.

[4] Ovchinnikov S, Park H, Kim DE, DiMaio F, Baker D (2018). Protein structure prediction using Rosetta in CASP12. Proteins: Structure, Function, and Bioinformatics.

[5] Greener JG, Kandathil SM, Jones DT (2019). Deep learning extends de novo protein modelling coverage of genomes using iteratively predicted structural constraints. Nature communications.

[6] Abramson J, Adler J, Dunger J, Evans R, Green T, Pritzel A, Ronneberger O, Willmore L, Ballard AJ, Bambrick J (2024). Accurate structure prediction of biomolecular interactions with AlphaFold 3. Nature.

[7] Jumper J, Evans R, Pritzel A, Green T, Figurnov M, Ronneberger O, Tunyasuvunakool K, Bates R, Žídek A, Potapenko A (2021). Highly accurate protein structure prediction with AlphaFold. nature.

[8] Krishna R, Wang J, Ahern W, Sturmfels P, Venkatesh P, Kalvet I, Lee GR, Morey-Burrows FS, Anishchenko I, Humphreys IR (2024). Generalized biomolecular modeling and design with RoseTTAFold All-Atom. Science.

[9] Lin Z, Akin H, Rao R, Hie B, Zhu Z, Lu W, Smetanin N, Verkuil R, Kabeli O, Shmueli Y (2023). Evolutionary-scale prediction of atomic-level protein structure with a language model. Science.

[10] Varadi M, Bertoni D, Magana P, Paramval U, Pidruchna I, Radhakrishnan M, Tsenkov M, Nair S, Mirdita M, Yeo J (2024). AlphaFold Protein Structure Database in 2024: providing structure coverage for over 214 million protein sequences. Nucleic acids research.

[11] Mirdita M, Schütze K, Moriwaki Y, Heo L, Ovchinnikov S, Steinegger M (2022). ColabFold: making protein folding accessible to all. Nature methods.

[12] Burley SK, Bhikadiya C, Bi C, Bittrich S, Chao H, Chen L, Craig PA, Crichlow GV, Dalenberg K, Duarte JM (2023). RCSB Protein Data Bank (RCSB. org): delivery of experimentally-determined PDB structures alongside one million computed structure models of proteins from artificial intelligence/machine learning. Nucleic acids research.

[13] Altschul SF, Madden TL, Schaffer AA, Zhang J, Zhang Z, Miller W, Lipman DJ (1997). Gapped BLAST and PSI-BLAST: a new generation of protein data base search programs. Nucleic Acids Res.

[14] Varadi M, Anyango S, Appasamy SD, Armstrong D, Bage M, Berrisford J, Choudhary P, Bertoni D, Deshpande M, Leines GD (2022). PDBe and PDBe-KB: Providing high-quality, up-to-date and integrated resources of macromolecular structures to support basic and applied research and education. Protein Science.

[15] Suzek BE, Wang Y, Huang H, McGarvey PB, Wu CH, Consortium, U (2015). UniRef clusters: a comprehensive and scalable alternative for improving sequence similarity searches. Bioinformatics.

[16] Kabsch W, Sander C (1983). Dictionary of protein secondary structure: pattern recognition of hydrogen-bonded and geometrical features. Biopolymers.

[17] McGuffin LJ, Bryson K, Jones DT (2000). The PSIPRED protein structure prediction server. Bioinformatics.

[18] Steinegger M, Meier M, Mirdita M, Vöhringer H, Haunsberger SJ, Söding J (2019). HH-suite3 for fast remote homology detection and deep protein annotation. BMC bioinformatics.

[19] Krivov GG, Shapovalov MV, Dunbrack RL (2009). Improved prediction of protein side-chain conformations with SCWRL4. Proteins: Structure, Function, and Bioinformatics.

[20] Hanson RM, Prilusky J, Renjian Z, Nakane T, Sussman JL (2013). JSmol and the Next-Generation Web-Based Representation of 3D Molecular Structure as Applied to Proteopedia. Israel Journal of Chemistry.

[21] Ribeiro AJM, Holliday GL, Furnham N, Tyzack JD, Ferris K, Thornton JM (2018). Mechanism and Catalytic Site Atlas (M-CSA): a database of enzyme reaction mechanisms and active sites. Nucleic acids research.

[22] Ward JJ, McGuffin LJ, Bryson K, Buxton BF, Jones DT (2004). The DISOPRED server for the prediction of protein disorder. Bioinformatics.

[23] Uziela K, Wallner B (2016). ProQ2: estimation of model accuracy implemented in Rosetta. Bioinformatics.

[24] Schmidtke P, Le Guilloux V, Maupetit J, Tufféry P (2010). Fpocket: online tools for protein ensemble pocket detection and tracking. Nucleic acids research.

[25] Jefferys BR, Kelley LA, Sternberg MJ (2010). Protein folding requires crowd control in a simulated cell. J Mol Biol.

[26] DeLano WL (2002). The PyMOL molecular graphics system.

[27] Robin X, Haas J, Gumienny R, Smolinski A, Tauriello G, Schwede T (2021). Continuous Automated Model EvaluatiOn (CAMEO)—Perspectives on the future of fully automated evaluation of structure prediction methods. Proteins: Structure, Function, and Bioinformatics.

[28] David A, Sternberg MJ (2023). Protein structure-based evaluation of missense variants: resources, challenges and future directions. Current Opinion in Structural Biology.

[29] Ittisoponpisan S, Islam SA, Khanna T, Alhuzimi E, David A, Sternberg MJE (2019). Can Predicted Protein 3D Structures Provide Reliable Insights into whether Missense Variants Are Disease Associated?. J Mol Biol.

[30] Pennica C, Hanna G, Islam SA, Sternberg MJ, David A (2023). A new web resource to predict the impact of missense variants at protein interfaces using 3D structural data: Missense3D-PPI. bioRxiv.

[31] Hanna G, Khanna T, Islam SA, David A, Sternberg MJ (2024). Missense3D-TM: Predicting the Effect of Missense Variants in Helical Transmembrane Protein Regions Using 3D Protein Structures. Journal of Molecular Biology.

[32] Van Durme J, Delgado J, Stricher F, Serrano L, Schymkowitz J, Rousseau F (2011). A graphical interface for the FoldX forcefield. Bioinformatics.

